# Sympathetic Activation in Hypertensive Chronic Kidney Disease – A Stimulus for Cardiac Arrhythmias and Sudden Cardiac Death?

**DOI:** 10.3389/fphys.2019.01546

**Published:** 2020-01-14

**Authors:** Márcio Galindo Kiuchi, Jan K. Ho, Janis Marc Nolde, Leslie Marisol Lugo Gavidia, Revathy Carnagarin, Vance B. Matthews, Markus P. Schlaich

**Affiliations:** ^1^Dobney Hypertension Centre, School of Medicine – Royal Perth Hospital Unit/Medical Research Foundation, The University of Western Australia, Perth, WA, Australia; ^2^Departments of Cardiology and Nephrology, Royal Perth Hospital, Perth, WA, Australia; ^3^Neurovascular Hypertension & Kidney Disease Laboratory, Baker Heart and Diabetes Institute, Melbourne, VIC, Australia

**Keywords:** chronic kidney disease, sympathetic nervous system, hypertension, left ventricular hypertrophy, sudden cardiac death, ventricular remodeling, renal denervation

## Abstract

Studies have revealed a robust and independent correlation between chronic kidney disease (CKD) and cardiovascular (CV) events, including death, heart failure, and myocardial infarction. Recent clinical trials extend this range of adverse CV events, including malignant ventricular arrhythmias and sudden cardiac death (SCD). Moreover, other studies point out that cardiac structural and electrophysiological changes are a common occurrence in this population. These processes are likely contributors to the heightened hazard of arrhythmias in CKD population and may be useful indicators to detect patients who are at a higher SCD risk. Sympathetic overactivity is associated with increased CV risk, specifically in the population with CKD, and it is a central feature of the hypertensive state, occurring early in its clinical course. Sympathetic hyperactivity is already evident at the earliest clinical stage of CKD and is directly related to the progression of renal failure, being most pronounced in those with end-stage renal disease. Sympathetic efferent and afferent neural activity in kidney failure is a crucial facilitator for the perpetuation and evolvement of the disease. Here, we will revisit the role of the feedback loop of the sympathetic neural cycle in the context of CKD and how it may aggravate several of the risk factors responsible for causing SCD. Targeting the overactive sympathetic nervous system therapeutically, either pharmacologically or with newly available device-based approaches, may prove to be a pivotal intervention to curb the substantial burden of cardiac arrhythmias and SCD in the high-risk population of patients with CKD.

## Introduction

The prevalence of chronic kidney disease (CKD) is ∼8–12% in most countries becoming a significant public issue across the globe, and it can progress to end-stage renal disease (ESRD) and renal replacement therapy (RRT). In these patients, the most typical cause of death is by far cardiovascular (CV) complications. Congestive heart failure (HF) and cardiac arrhythmias, in particular, are highly prevalent and a common reason for hospitalization (Kiuchi and Mion, 2016; Kusumoto et al., 2018). As the renal impairment has been considered an independent hazard for sudden cardiac death (SCD), several cohort studies and clinical trials have used it as a well-defined endpoint (Kiuchi and Mion, 2016; Kusumoto et al., 2018).

## Sudden Cardiac Death, Chronic Kidney Disease, and Epidemiology

### Chronic Kidney Disease

Clinical trials evaluating the effectiveness of automatic implantable cardioverter-defibrillators (ICDs) have demonstrated the high hazard of SCD in the CKD population. These findings are supported by the 2018 United States Renal Data System (USRDS) (Table 1; United States Renal Data System, 2018).

**TABLE 1:** Prevalence of HF, SCD, and VA and annual incidence of cardiovascular procedures.

**% Patients**						
	**#Patients**	**Overall**	**66–69**	**70–74**	**75–84**	**≥85**
**Heart failure**						
Non-CKD	1,086,232	6.1	3.1	4.3	7.2	13.3
Any CKD	175,840	25.9	18.3	20.1	25.7	36.1
**SCD/VA**						
Non-CKD	1,086,232	1.4	1.0	1.4	1.8	1.8
Any CKD	175,840	4.1	3.4	3.9	4.4	4.3
**ICD/CRT-D**						
Non-CKD	66,426	0.6	0.6	0.8	0.6	0.3
Any CKD	45,552	1.0	1.5	1.4	1.1	0.6

*CKD, chronic kidney disease; CRT-D, cardiac resynchronization therapy with defibrillator; HF, heart failure; ICD, implantable cardioverter-defibrillator; SCD, sudden cardiac death, VA, ventricular arrhythmias. Adapted from 2018 United States Renal Data System annual data report (United States Renal Data System, 2018).*

**FIGURE 1:**
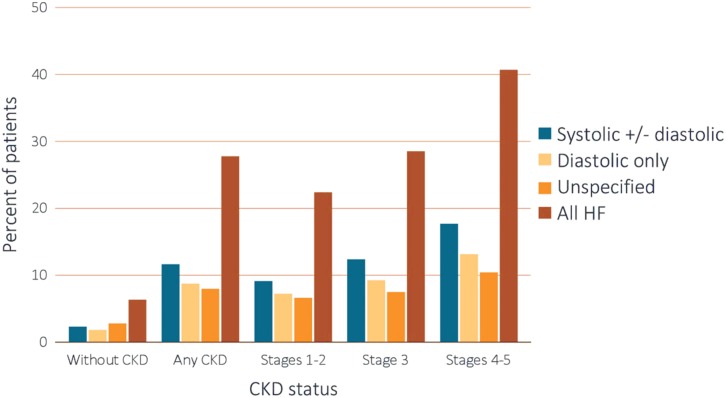
Heart failure in healthy and CKD subjects. CKD, chronic kidney disease; HF, heart failure. Adapted from 2018 United States Renal Data System annual data report (United States Renal Data System, 2018). Reproduced with permission.

On optimized pharmacological therapy, every 10 mL/min/1.73 m^2^ glomerular filtration rate fall accounted for an increase of SCD risk by 17% (Goldenberg et al., 2006). Given the relevance and prognostic implications of HF in this population, the existence or non-existence of LV systolic dysfunction (i.e., “systolic” HF with reduced ejection fraction, “diastolic” HF with preserved ejection fraction, or unspecified) was meticulously examined (Figure 1). All types of HF were more typical amongst CKD subjects in comparison to those without CKD. The proportional ratio of individuals having CKD and systolic HF concomitantly was demonstrated to be greater than for diastolic HF; also, the prevalence increased with CKD progression (United States Renal Data System, 2018).

**FIGURE 2:**
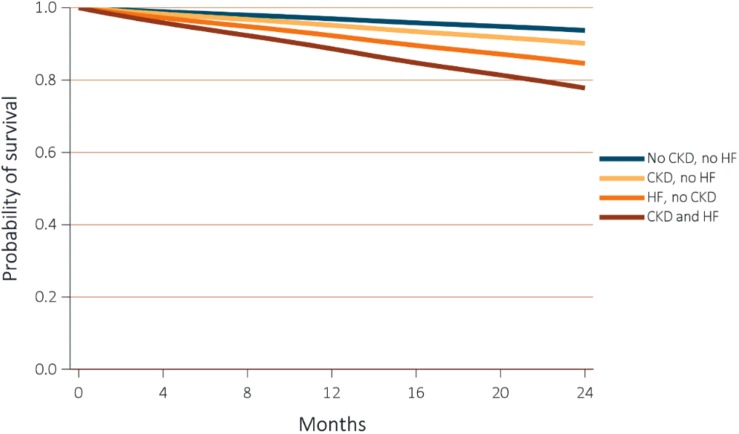
Adjusted survival of subjects by CKD and heart failure status. Survival was adjusted for age, sex, race, diabetic, and hypertension status. CKD, chronic kidney disease; HF, heart failure. Adapted from 2018 United States Renal Data System annual data report (United States Renal Data System, 2018). Reproduced with permission.

The “Comparison of Medical Therapy, Pacing, and Defibrillation in Heart Failure Trial” showed late stages HF patients with intraventricular electrical conduction disorders (e.g., bundle branch block) had significant reductions in death and hospitalization rates after cardiac resynchronization therapy (Bristow et al., 2004). In addition, existing renal dysfunction raised the harm for SCD by 67% during the 16-months of follow-up (Saxon et al., 2006). HF reduced survival rates amongst CKD and non-CKD groups (Figure 2), although this was more pronounced in the former (*p*-value for interaction <0.0001) (United States Renal Data System, 2018). After 2 years, the adjusted survival probability was 77.8% for patients with concomitant HF and CKD, 84.6% for HF subjects without CKD, 90.2% for non-HF individuals with CKD, and 93.7% for those without CKD and HF (United States Renal Data System, 2018). Analyzing the 2-year survival of patients with HF according to their CKD stages the survival rates fell with further progression of renal disease (stages 1–2 = 70.2%, 3 = 65.8%, and 4–5 = 55.7%, respectively). A similar 2-year survival trend was observed in patients with CKD who presented with ventricular arrhythmias (VAs). Survival rates of healthy individuals (86%) were higher than those for patients with CKD (68.8%). Further deterioration of kidney function was associated with lower survival rates (Stage 1–2 = 75.4%, Stage 3 = 68.7%, and Stage 4–5 = 57.9%) (United States Renal Data System, 2018). Furthermore, a correlation between SCD and renal damage was shown in non-HF individuals with moderate CAD (Deo et al., 2008; Pun et al., 2009).

Several investigations have attempted to outline SCD hazard amongst CKD subjects taking into account the influences of confounding prevalent CV diseases. A community study, including 4,465 participants without any previous history of myocardial infarction (MI) or HF demonstrated that the SCD incidence was 2.5 folds higher in those who had advanced CKD (Saxon et al., 2006). The SCD hazard was doubled in participants with “pre-clinical renal disease” (Deo et al., 2010) (defined as estimated glomerular filtration rate: eGFR >60 mL/min/1.73 m^2^ and Cystatin C level ≥1.0 mg/L) matched to those with unaltered renal function (defined as eGFR >60 mL/min/1.73 m^2^ and Cystatin C level <1.0 mg/L). This suggests that even minor drops in the eGFR may raise SCD risk, particularly in the elderly (Deo et al., 2010).

### End-Stage Renal Disease

In the current context, most CV deaths reported in ESRD are attributed to SCD (Levey et al., 1998). In this population, arrhythmic deaths and MI represent 22% of all deaths (Herzog et al., 2005). In a cohort of 1,041 hemodialysis patients, 658 deaths were reported over an 8-year follow-up period. Amongst them, 146 deaths were attributed to SCD (rate of 1.8% per year) (Parekh et al., 2008). Similarly, a significant frequency of SCD at 5 years of follow-up (4.9% per year) was reported by a Chinese prospective trial comprising 230 participants, in which reduced LVEF and widened pulse pressure were considered predictors of SCD (Wang et al., 2010).

Congestive HF is commonly characterized by sustained renin-angiotensin-aldosterone system (RAAS) and sympathetic nervous system (SNS) overactivation, promoting higher Na^+^ and water retention with increased vascular tone and structural cardiac, renal and vascular remodeling. On the heart level, this resulting increase in LV and atrial pressure stimulates natriuretic peptide synthesis and secretion. Moreover, natriuretic peptides exert a key function in regulating blood pressure and extracellular fluid volume (Volpe et al., 2016). In non-adjusted and completely adjusted analyses, N-terminal pro-hormone of brain natriuretic peptide (NT-proBNP) correlated strongly with SCD hazard (Kruzan et al., 2016). When assessed as a continuous parameter, SCD hazard rose by 27% at every 2-fold NT-proBNP growth. In categorical analyses, SCD hazard was 3-fold greater in the NT-proBNP top tertile (>7,350 pg/mL) in comparison to its bottom tertile (<1,710 pg/mL). There was a trend for an association of elevated troponin I (cTnI) levels with a heightened risk of SCD in entirely adjusted models. Sensitivity assessments by comparing hazard analyses demonstrated analogous outcomes. Enhancement in hazard prediction by cardiac biomarkers addition to typical hazard factors seemed to be higher with NT-proBNP (concordance statistic for 3-year hazard: 0.810; 95% confidence interval, 0.757–0.864; and continuous net reclassification improvement: 0.270; 95% confidence interval, 0.046–0.495) compared to cTnI (Kruzan et al., 2016). The presence of elevated levels of cardiac biomarkers has not only been associated with higher SCD occurrence but also with reversible myocardial stunning. An observational cohort study of 70 prevalent hemodialysis patients found that myocardial stunning was common during dialysis (64%) and was related to increased relative mortality and development of low LVEF after 1 year (Burton et al., 2009). Even though a reduced LVEF in the non-dialysis population is a hazard element for SCD, it does not appear to be as relevant as in the ESRD population. In a review of 80 ESRD deaths that occurred secondary to SCD, a LVEF ≤35% was only evident in one-fourth of the patients (Bleyer et al., 2006).

The prevalence of HF, SCD and the occurrence of VAs and ICD/CRT-D implantation were analyzed in ESRD patients on RRT in the 2018 USRD annual report, and the findings are summarized in Table 2 (United States Renal Data System, 2018). The 2-year survival rates of ESRD adult patients with and without HF were 66.0 and 93.4%, respectively. The respective rates in the presence and absence of SCD and VAs were 55.3 and 77.2%. When ICD/CRT-D were implanted in these patients, their 2-year survival rate was inferior (48.1%) compared to subjects who did not undergo device implantation (62.9%) (United States Renal Data System, 2018). This, however, could perhaps be attributed to the severity of the underlying CV disease, as ICD/CRT-D procedures are performed mostly in late stages of HF.

**TABLE 2:** Prevalence of HF, SCD, and VA and annual incidence of cardiovascular procedures in End-stage renal disease individuals.

**% Patients**					
	**#Patients**	**Overall**	**66–69**	**70–74**	**≥75**
**Heart failure**					
Hemodialysis	218,720	40.4	28.3	38.5	44.7
Peritoneal dialysis	22,023	28.3	19.9	27.6	32.7
Transplant	75,313	14.4	6.0	12.4	19.5
**SCD/VA**					
Hemodialysis	218,720	4.8	2.9	4.6	5.7
Peritoneal dialysis	22,023	4.6	2.1	4.4	5.8
Transplant	75,313	2.0	0.6	1.6	3.1
**ICD/CRT-D**					
Hemodialysis	88,377	0.9	0.9	1.0	1.0
Peritoneal dialysis	6,181	1.1	0.6	1.2	1.3
Transplant	10,851	0.8	0.1	0.7	0.9

*CRT-D, cardiac resynchronization therapy with defibrillator; HF, heart failure; ICD, implantable cardioverter-defibrillator; SCD, sudden cardiac death; VA, ventricular arrhythmias. Adapted from 2018 United States Renal Data System annual data report. Adapted from 2018 United States Renal Data System annual data report (Kusumoto et al., 2018).*

**FIGURE 3:**
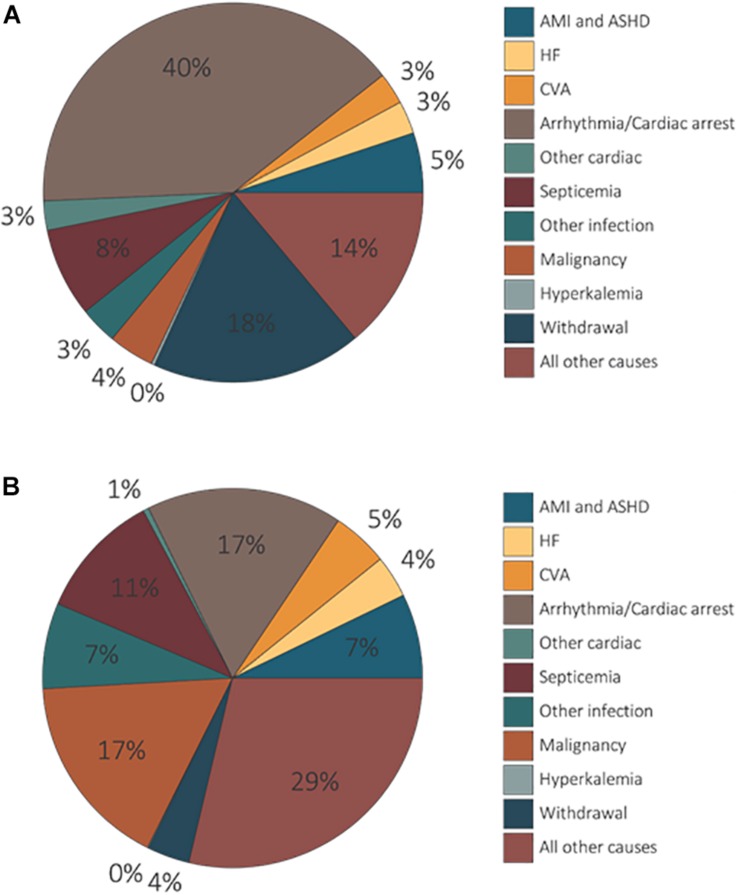
Unadjusted percentages of deaths by cause, modality amongst dialysis patients and transplant recipients. Dialysis patients’ data exclude missing/unknown causes of death **(A)**. Transplant patients’ denominator excludes missing/unknown causes of death **(B)**. AMI, acute myocardial infarction; ASHD, atherosclerotic heart disease; CHF, congestive heart failure; CVA, cerebrovascular accident (United States Renal Data System, 2018). Reproduced with permission.

Cardiac arrhythmia/arrest accounted for ≈40 and 17% of identified causes of death amongst individuals on dialysis and transplant recipients, respectively (Figure 3; United States Renal Data System, 2018). The cause of death was not available or unidentified for 27 and 74% of dialysis patients and transplant participants recruited for the study, respectively. CV causes (arrhythmias, cardiac arrest, congestive HF, acute MI, and coronary atherosclerosis) were responsible for 48 and 28% of deaths amongst subjects on dialysis or transplant receivers, respectively (United States Renal Data System, 2018).

In the context of ESRD CV disease is known to contribute to rising mortality rates in the short and long-term. USRDS data (Herzog et al., 1998) demonstrated, after 1 year of follow-up, a mortality rate of 60% in patients on long-term dialysis post-acute MI (United States Renal Data System, 2018). In general, the presence of CV disease in ESRD patients significantly worsens their survival rates. Finally, the robust relationship between SCD and ESRD also spreads to the pediatric population. A retrospective assessment of USRDS data comprising ∼1,400 deaths amongst ESRD individuals aged up to 30 years showed that cardiac arrest and arrhythmias accounted for most deaths associated with cardiac causes (>2%/year) (Parekh et al., 2002). These findings indicate that mechanisms other than those associated with CAD and/or HF prompt fatal arrhythmias in subjects with ESRD.

## Pathophysiologic Mechanisms Contributing to High Risk for Arrhythmias

Sudden cardiac death pathophysiology has been described as multifaceted; combinations of various factors can result in electrical conduction volatility and VAs, followed by hemodynamic collapse. Understanding this mechanism may assist in identifying the time point at which the interaction between a prompting incident and a present source is risky. Remodeling of the structure and electrophysiologic properties of the heart, fibrosis, coronary artery calcification, autonomic imbalance, and volume and electrolyte changes are all considered contributors to this scenario.

### Structural Remodeling

Renal impairment provokes cardiac remodeling including LV hypertrophy (LVH) and its fibrosis. Clinical evidence revealed an independent correlation between LVH and CKD in subjects with mild to moderate reduction in eGFR (Levin et al., 1999; Paoletti et al., 2005; Moran et al., 2008; Cerasola et al., 2010). Notably, the frequency of LVH increases with worsening of renal function. Furthermore, fibrosis and non-ischemic cardiomyopathy were demonstrated by magnetic resonance imaging (MRI) in dialysis patients (Mark et al., 2006). Hypertension, diabetes mellitus, and anemia were common co-morbidities and may partially explain the LV remodeling that occurs (Schroeder et al., 1997; Hunter and Chien, 1999; Cioffi et al., 2011). Mechanistically, activation of growth factors, proto-oncogenes, and cytokines, together with increased norepinephrine and angiotensin II plasma concentrations are likely contributors through their well-defined effects on cardiac structure, from hypertrophy to fibrosis and apoptosis (Mall et al., 1990; Amann et al., 1998). A higher hazard of sustained ventricular tachycardia (VT) and SCD susceptibility has been associated with those structural changes *per se* (Haider et al., 1998; Yan et al., 2006; Schmieder et al., 2007; Roes et al., 2009; Reinier et al., 2011).

Chronic kidney disease is also linked to peripheral vascular disease (Pai and Giachelli, 2010; Briet et al., 2011; Hu et al., 2011; Shroff and Shanahan, 2011). The reduction of vascular elasticity is correlated with worsening eGFR and endothelial dysfunction. An inadequate endothelium-dependent vasodilator response can already be detected in mild renal disease (Perticone et al., 2004, 2010). As a consequence of vessel remodeling and sclerosis, coronary perfusion reserve is affected and can raise the risk of ischemic events (Kingwell et al., 2002) and arrhythmias. In ESRD, vascular remodeling is more apparent as calcium-phosphate accumulation, which may additionally provoke vascular integrity impairment (Schlieper et al., 2010). Excessive phosphate levels and enhanced calcium-phosphate product have been demonstrated to raise, SCD risk from 20 to 30% (Ganesh et al., 2001).

### Electrophysiological Alterations

Structural alterations can affect the myocardial electrophysiology. Myocardial fibrosis changes the tissue structure slowing down stimulus conduction through the fibers (Waldo et al., 1983), which can sustain re-entrant arrhythmias (e.g., VT) (Yan et al., 2006; Schmidt et al., 2007; Roes et al., 2009). Altered cardiac conduction can retard the activation of the ventricles and thereby, in the last segment of the QRS complex, produce late action potentials. They are low amplitude signals and were identified in 25% of patients on RRT (Morales et al., 1998). Also, the non-homogeneous retrieval of the excitability of the ventricles was reported by studies evaluating QT interval dispersion; it is mainly increased in the post-dialysis period (Lorincz et al., 1999; Morris et al., 1999; Patel et al., 2011) and may reflect a higher vulnerability to VAs (Kiuchi and Mion, 2016). Post-evaluation by a 24-h Holter monitoring revealed that >90% of RRT patients presented with abnormal T-wave alternans (TWA) throughout 24 h (Secemsky et al., 2011) and that dialysis sessions could provoke alterations in TWA (Friedman et al., 2007; Green et al., 2012). A study comprising 48 participants demonstrated that TWA rose throughout dialysis sessions returning to basal levels after dialysis. However, the investigators did not find any correlation with electrolyte levels, LV structure and function, major adverse CV events, or mortality (Green et al., 2012).

Malignant arrhythmias and SCD may also be associated with dialysis frequency in ESRD individuals. After a lengthy interdialytic period, a failure to keep the desirable homeostasis may occur predisposing patients to harmful events (Kiuchi and Mion, 2016). Undeniably, VAs and SCD often occur on Mondays and Tuesday’s after the long dialysis-free interval over the weekend and within 12 h subsequent to the HD sessions beginning (Bleyer et al., 1999, 2006; Perl and Chan, 2006; Foley et al., 2011; Kumar et al., 2016) suggesting that BP changes and variations of electrolyte- and volume- homeostasis can provoke arrhythmias.

Myocardial scars create areas of heterogeneous electricity conveyance predisposing to re-entrant arrhythmias, specifically in the context of renal dysfunction initiated either automatically or elicited by different regions within the myocardium (Brotman et al., 2010). The association of ESRD with increased sympathetic nerve discharge rates and the susceptibility of abnormal rhythms to adrenergic activity have been shown in some human studies. These are mediated partially by afferent discharges arising from the kidney (Converse et al., 1992; Svarstad et al., 2001; Hausberg and Grassi, 2007) providing a potential basis for a high incidence of ventricular ectopic beats (VEBs) in >75% of patients with ESRD for the period of and post to dialysis sessions (Gruppo Emodialisi E Patologie Cardiovascolari, 1988). Sympathetic activity in these subjects is perhaps more likely than in other patient cohorts to results in adverse consequences (Zoccali et al., 2002).

## Sympathetic Overactivity in Hypertension and CKD

In patients with CKD, sympathetic hyperactivity raises the CV risk and exerts a critical role in increasing blood pressure. Also, sympathetic overdrive can already be found in the earliest phases of CKD (Grassi, 2009, 2010; Paton and Raizada, 2010). In both hypertension and CKD, several mechanisms contribute to sympathetic excitation, including reflex and neuro-humoral pathways (McGrath et al., 1978; Grassi, 2009, 2010). Sympathetic overactivity has been correlated to CKD evolvement and can be triggered by several types of renal damage (McGrath et al., 1978; Neumann et al., 2004; Schlaich et al., 2009a; Grassi et al., 2012). Afferent and efferent nerves surrounding renal vessels, tubules, the pelvis, and glomeruli supply renal innervation. It facilitates the interconnection within the neuro-cardio-renal axis (Drukker et al., 1987; Barajas et al., 1992; Kumagai et al., 2012; Mahmoodi et al., 2012; Mulder et al., 2013) through a two-way neural path to convey afferent and efferent sympathetic impulses to and from the brain, respectively (Guertzenstein and Silver, 1974; Campese and Kogosov, 1995; Garthwaite and Boulton, 1995; Ye et al., 1997; Zanzinger, 2002; Kimura et al., 2005; Kumagai et al., 2012). Increased sympathetic tone, via renal efferent nerves, modifies tubular reabsorption of Na^+^ and H_2_O with subsequent fluid retention, renal blood flow decrease, and RAAS stimulation (DiBona and Kopp, 1997; Krum et al., 2009; Schlaich et al., 2009a, b; Grassi et al., 2012). The central integration of afferent renal stimulus to regulate primary sympathetic discharge completes the feedback loop (Campese and Kogosov, 1995; DiBona and Kopp, 1997; Ye et al., 1997; Kumagai et al., 2012).

**FIGURE 4:**
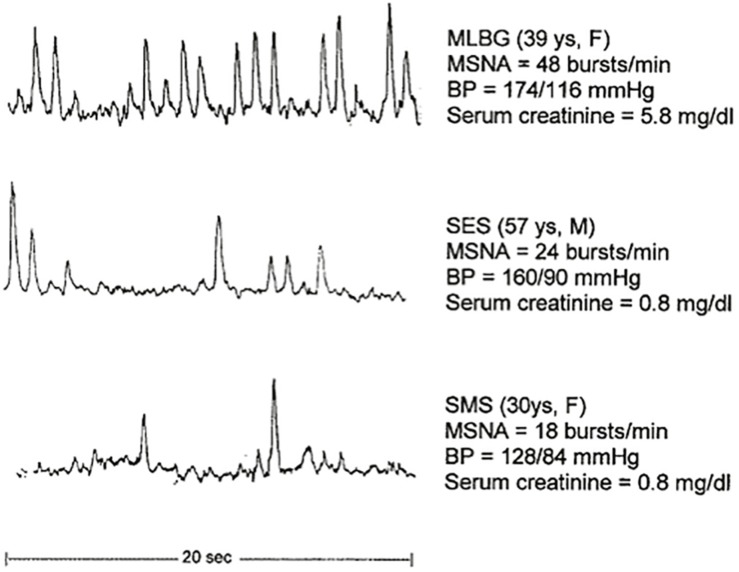
MSNA recordings in individuals with mild CKD, and hypertensive and normotensive individuals. BP, blood pressure; CKD, chronic kidney disease; MSNA, muscle sympathetic nerve activity (Tinucci et al., 2001). Reproduced with permission.

Hypertensive states, other CV diseases, CKD and ESRD, are often characterized by augmented renal sympathetic nerve activity (RSNA) (Lundin et al., 1984; Grassi et al., 1998; Esler et al., 2003; Campese et al., 2006) as demonstrated by high levels of muscle sympathetic nerve activity (MSNA) documented in every stage of human hypertension (Grassi et al., 1998). High levels of renal norepinephrine spillover have been described in human hypertension (Esler et al., 2003) and increased renal sympathetic nerve firing rates in preclinical models (Lundin et al., 1984). Meta-analyses have revealed that worsening in eGFR is an independent CV hazard (Mahmoodi et al., 2012) and that sympathetic activation is linked to poorer CV outcomes (Zoccali et al., 2002; Grassi, 2009, 2010; Grassi et al., 2012). There is clear data that hypertensive patients with mild renal damage have significant elevated MSNA in comparison to hypertensive individuals with normal renal function and to normotensive populations (Figure 4; Tinucci et al., 2001). A close relation between sympathetic activation and gradual renal function decline is evident.

Afferent sensory renal nerves are crucial to modulate SNS activity, as has been elegantly shown in studies conducted in ESRD and post-renal transplant patients. Increased SNS activity persists in ESRD patients despite hemodialysis and even after renal transplantation due to the remaining native kidneys (Converse et al., 1992). Indeed, BP and MSNA can be normalized by removal of the native kidneys. Consequently, focusing on the renal sympathetic nerves as a therapeutic target is a reasonable approach to treat hypertension and states of renal damage to prevent the progression of renal impairment (Barnett et al., 2014).

Accumulating evidence suggests a potential role of gut dysbiosis in the pathophysiological mechanisms involved in hypertension and CKD via the neuroendocrine regulation of immunity. The gut microbiome sustains intricate communication with vital organs (e.g., immune system, bone marrow, blood vessels, kidneys, and central and autonomic nervous system: ANS) to control cardiometabolic homeostasis (Bercik et al., 2011; Bravo et al., 2011; Karbach et al., 2016; Rooks and Garrett, 2016; Dinan and Cryan, 2017; Evenepoel et al., 2017; Josefsdottir et al., 2017). In addition to the endocrine effect, the gut microbiome also exerts a paracrine influence via its metabolites (Akchurin and Kaskel, 2015; Santisteban et al., 2015; Shankland and Jefferson, 2017; Carnagarin et al., 2019). Gut dysbiosis may play an important role in the onset and advance of CKD. Gut dysbiosis elicits immune-mediated inflammatory responses affecting regulatory brain nuclei and its relations to the SNS and thereby the kidneys (Carnagarin et al., 2019). This provokes a positive feedback loop where the resulting sympathoexcitation further promotes gut dysbiosis and increases gut permeability allowing the escape of toxic metabolites in the circulation as seen in CKD (Vanholder et al., 2003; Martinez et al., 2005; Jourde-Chiche et al., 2009; Vaziri, 2012; Sabatino et al., 2015).

## Therapeutic Targeting of SNS Activation

Given the great importance of SNS activation in hypertension and CKD and its close association with adverse CV consequences, including SCD, therapeutic targeting of the SNS at various levels is expected to confer clinical benefit. A conventional approach is the blockade of peripheral β-receptors, and more recently, direct interference with renal afferent and efferent nerves through RDN has shown promising results.

### β-Blockers

The anti-arrhythmic effectiveness of β-blockers is based on the competitive β-adrenoreceptor block of pro-arrhythmic properties mediated by the SNS, including decelerating of sinus frequency and probably the inhibition of additional Ca^2+^ discharge via the RyR2 channels. VEBs and VAs suppression, as well as SCD rate reduction, have been demonstrated with β-blockers particularly in HF (Krittayaphong et al., 2002; Prystowsky et al., 2012; Shinohara et al., 2017). β-blockers are considered efficient and safe anti-arrhythmic drugs and represent an established pharmacological anti-arrhythmic therapy. Generally, VAs management and SCD prevention utilise β-blockers as the pillar for their treatment (Priori et al., 2016).

### Renal Denervation

Renal denervation is a device-based technique to directly modulate efferent and afferent nerves connecting both kidneys and central integrative structures in the brain. In resistant hypertensive patients, a marked BP-lowering effect and a fast decrease in the activity of single fibre sympathetic nerve units were noticed post-RDN (Hering et al., 2013). Moreover, sympathetic overactivity and RAAS disruption are likely to benefit this population. RDN has been proved to be safe in all studies carried out thus far, and may be of specific benefit in hypertensive CKD subjects (Hering et al., 2012; Kiuchi et al., 2013; Luo et al., 2013; Schlaich et al., 2013). Similarly, ESRD subjects with poorly controlled hypertension had a continuous systolic office BP-lowering effect and an important fall in MSNA over 12-months post-RDN, without safety concerns (Schlaich et al., 2013).

Aside from the improved BP control, the regression of hypertensive target organ damage is considered a good indicator of therapeutic efficiency. Indeed, structural and functional changes were noticed as assessed by echocardiography post-RDN. This effect was also observed in 83% of the participants who were classified as “non-responders,” as their BP-lowering effect was less than 10 mmHg (Brandt et al., 2012). Direct modulation of SNS activity may have benefits beyond its impact on BP with implications for high-risk populations (Brandt et al., 2012; Kiuchi and Mion, 2016).

In another study (Mahfoud et al., 2014) 72 subjects with resistant hypertension underwent cardiac-MRI (55 of them underwent RDN, and 17 were controls) prior to and 6 months after RDN. A remarkable BP-lowering effect in both systolic and diastolic BP (∼22/8 mmHg), as well as a reversal of the left ventricular remodeling (LV mass index regression of 7.1%), were reported post-RDN. On the other hand, control subjects did not present any variation. After RDN, average LVEF significantly improved by ≈7%. In this study, LV circumferential strain substituted diastolic function. Patients who were subjected to RDN had their circumferential strain significantly improved by 21%, while control subjects did not experience this effect.

Doltra et al. (2014) prospectively assessed 23 resistant hypertensive patients who had undergone cardiac-MRI and RDN. RDN led to a decrease in LVMI regardless of the BP-lowering effect, suggesting that RDN may additionally reduce interstitial fibrotic tissue in the myocardium, regarding absolute collagen matter, because if the observed LVMI regression was exclusively owing to a reversal of myocyte hypertrophy, extracellular volume fraction would be expected to increase (Doltra et al., 2014). Whether this has a potential effect on the prognosis and the event reduction remains unknown. Besides, Perlini et al. (2005) did previously demonstrate in rodents that interstitial fibrosis in the myocardium provoked by hypertension improved after α-adrenergic block or sympathectomy.

More recently, McLellan et al. (2015) collected 24-h ambulatory-blood-pressure (ABPM), echocardiograms, cardiac-MRI and electrophysiological studies in 14 subjects presenting with resistant hypertension prior to and 6 months post-RDN. After RDN, the average ABPM was reduced, while overall conduction velocity rose considerably, and conduction interval was reduced. Also, changes in conduction velocity and variations in average ABPM correlated positively. In an ovine model, those with chronic hypertension had left atrial remodeling on diverse time-domains and a strong association between electrophysiology properties and structure involved in the remodeling flow. These successive morphological modifications were linked to conduction abnormalities and resulted in greater atrial fibrillation inducibility and duration. Immediate antihypertensive therapy commencement may avoid the development of substrates able to maintain atrial fibrillation (Lau et al., 2010). A marked reduction in MRI derived LV mass and diffuse ventricular fibrosis was also observed (McLellan et al., 2015), in keeping with studies discussed above.

Likewise, one hundred consecutive resistant hypertensive subjects who were subjected to RDN and experienced an average office systolic BP fall >10 mmHg at 6 month post-RDN were studied by Dorr et al. (2015). Cardiac extracellular matrix and CV fibrotic tissue reabsorption were assessed by different pro-peptide types before RDN and at 6 month follow-up through blood samples. A substantial office systolic BP drop was reported 6 months post-RDN. At this stage, the serum levels of pro-peptides were remarkably reduced compared to baseline in participants with an intensified collagen turnover. These results point to possible beneficial RDN effects on CV fibrosis in cohorts who have hypertensive heart disease and cardiac fibrosis.

Animal studies found sympathetic activation mitigation by RDN as assessed by renal catecholaminergic content reduction (Mahfoud et al., 2015). Moreover, RDN importantly improved LV longitudinal strain, reduced end-systolic volume and cardiac fibrosis, enhancing cardiac performance. Remarkably, neprilysin activity reduction and brain natriuretic peptide (BNP) level increases were shown after RDN (Ceia et al., 2002). In congestive HF, the excessive cardiac volume provokes BNP and atrial natriuretic peptide release, both of which have diuretic and cardioprotective effects (Stevens et al., 1996; da Silva and Aguiar, 2017). Heightened neprilysin activity was uncovered to worsen LV dysfunction, as it enzymatically degrades natriuretic and other bioactive peptides; and it is related to unfavorable outcomes. Therapeutically, HF populations have enormously benefited from neprilysin inhibition by sacubitril/valsartan (McMurray et al., 2014). RDN induced neprilysin inhibition may, therefore, contribute to its apparent cardioprotective effects.

While these findings are promising and suggest that sympatho-inhibitory therapeutic approaches may have substantial therapeutic benefit, further in-depth investigation of how RDN may reduce the burden of HF and SCD in CKD is warranted as are larger-scale studies to confirm results obtained predominantly in selected cohorts without appropriate controls.

**FIGURE 5:**
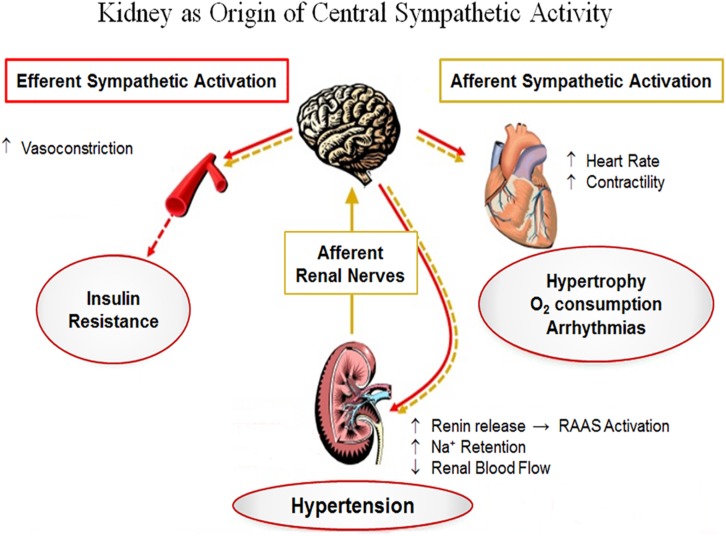
Numerous types of renal injury can trigger increased afferent signaling, which is centrally integrated leading to increased sympathetic discharges to various organs, including the kidneys, and thereby stimulating renin secretion, Na^+^ retention, and vasoconstriction. The increased sympathetic output is also pointed toward other relevant organs (e.g., the heart and peripheral vessels), which can result in adverse outcomes. RAAS, renin-angiotensin-aldosterone system; Na^+^, sodium; O_2_, oxygen. Adapted from Schlaich et al. (2009c). Reproduced with permission.

It is recognized that CKD progression can be slowed by a moderate control of BP. While RDN has been used predominantly to treat resistant hypertensive patients (Figure 5; Symplicity et al., 2010; Krum et al., 2014), it is likely of beneficial value in CKD. Indeed, RDN has demonstrated GFR improvement (Kiuchi et al., 2013, 2014; Delacroix et al., 2014) and reduction of albuminuria (Kiuchi et al., 2013, 2014; Ott et al., 2014; Schmieder et al., 2014), although these studies are limited by a short follow up period.

Kiuchi et al. (2016a) reported cardiac structural and functional enhancement, which were associated with eGFR improvement in a resistant hypertensive cohort with CKD at follow-up 6 month post-RDN. In this study, RDN improved echocardiographic parameters in patients with or without LVH (Kiuchi et al., 2016b). RDN was crucial in a series of cases comprising patients who had dilated cardiomyopathy and continuous episodes of VAs, as it impressively reduced the number of these arrhythmias and therefore the ICD shocks (Ukena et al., 2012). Growing data both from different case series (Remo et al., 2014; Armaganijan et al., 2015) and from a multicentre registry have shown favorable outcomes for anti-arrhythmic effects associated with RDN (Ukena et al., 2016). The limited data currently available suggest that RDN could be especially beneficial for HF subjects with VAs who are unresponsive to maximum β-blocker therapy and unsuitable for VT catheter-ablation. Alternatively, RDN may have an adjuvant role for those who will be subjected to catheter-ablation for refractory VAs.

In a cohort of HF subjects, NT-proBNP levels were reduced by RDN, which was well tolerated and did not worsen the function of the heart and the kidneys (Hopper et al., 2017). Also, the proportion of refractory VAs and ICD shocks plunged post-RDN in another cohort with late stages of HF and CKD (Kiuchi et al., 2017).

**FIGURE 6:**
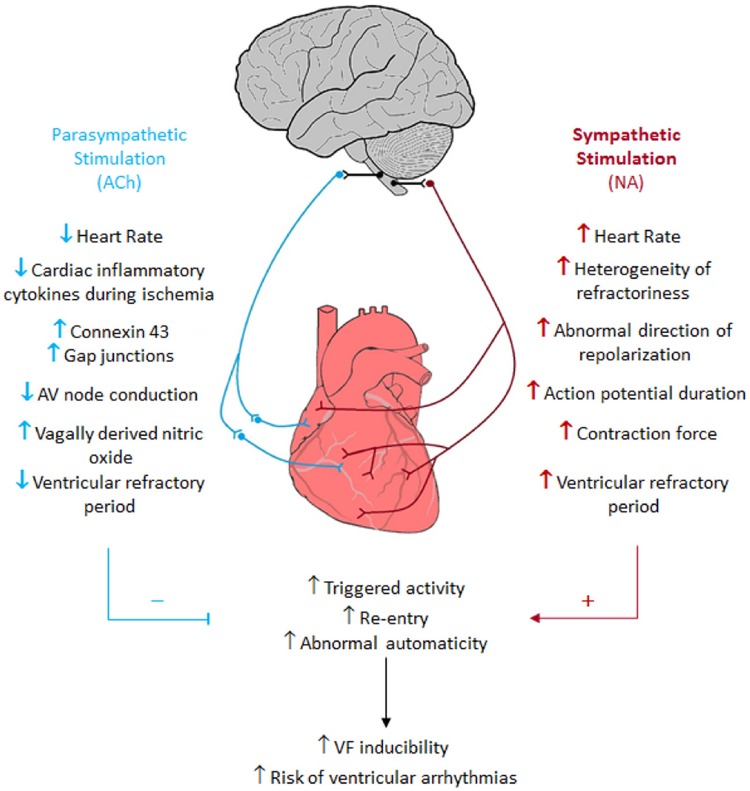
Sympathetic and parasympathetic nervous system cardiac arrhythmogenic effects. Blue = parasympathetic nervous system. Red = sympathetic nervous system. Black = Connections between the brain and the parasympathetic and sympathetic nervous systems. Up arrows = increase. Down arrows = decrease. Redline and + = provoke these events. Blue line and − = inhibit these events (Franciosi et al., 2017). Reproduced with permission.

The ANS imbalance plays a crucial role in advanced HF setting, which *per se* triggers and maintains VAs (Fukuda et al., 2015; Zipes, 2015). The hypersympathetic drive directed toward the heart can worsen other pre-existing conditions that contribute to VAs (e.g., ischemia, underlying rhythm and dilated cardiomyopathy) (Shen and Zipes, 2014; Fukuda et al., 2015). Unraveling the exact role of the ANS in the pathogenesis of VA will be rlevant for prevention and treatment of those arrhythmias (Ng, 2016; Franciosi et al., 2017; Figure 6).

Renal denervation may blunt sympathetic overactivity and arrhythmogenic foci, thereby potentially reducing arrhythmogenic activity. Preclinical data have shown remodeling of the stellate ganglion (SG) and the brain stem ∼2 months post-RDN, likely medaited by afferent renal nerve signaling interruption (Tsai et al., 2017). Also, a reduction of ^18^FDG- uptake, SG sympathetic traffic and atrial tachyarrhythmias were correlated to neural modifications of these structures. Therefore, such remodeling could at least in part explain the antiarrhythmic effects of RDN (Tsai et al., 2017). Even 1 year following a trauma, the central and peripheral nervous system may continue to impact on the damaged site and in distant zones of the brain (Bramlett and Dietrich, 2007). These gradual changes possibly underpin specific lasting effects following the primary damage, such as those provoked by RDN. In cats, roughly 10% of neurons located in renal sympathetic fibers arise from the ganglia composing the thoracic chain (Meckler and Weaver, 1984). Due to these interconnections, SG cell death may occur retrogradely by RDN. This is supported by data showing that certain colorants applied to renal nerves resulted in bright labeling of the sympathetic cells located in the para- and pre-vertebral ganglionic chains (Ferguson et al., 1986; Gattone et al., 1986; Sripairojthikoon and Wyss, 1987). Usually, sympathetic preganglionic fibers reaching both SG run through the cervicothoracic spinal cord (Pilowsky et al., 1992), which hugely increases interconnection possibilities between these fibers and those arising from the kidneys. It is also possible that several different routes (i.e. carotid sinus) contribute with collapse between synapses (Tsai et al., 2017), as afferent renal nerve ganglionic cells situated in lumbar and thoracic dorsal root ganglia of the spinal cord also connect to hypothalamic nuclei–specifically the posterior and lateral areas, and the locus ceruleus (Campese and Kogosov, 1995; Jansen et al., 1995). Together these data suggest that long-term RDN results may possibly occur due to remodeling of crucial brainstem sites and both SG.

## Conclusion and Future Research Directions

Sympathetic nervous system activation is a key feature of CKD and ESRD and causally linked to adverse cardiac consequences. The renal nerves, in particular, appear to be essential mediators and can now be targeted selectively. Data from small and mostly uncontrolled studies in relevant cohorts support the concept that targeting the renal nerves directly may confer benefit beyond blood pressure lowering. This therapeutic approach may beneficially impact on the CV sequelae commonly encountered in CKD and ESRD such as SCD and VAs. Clearly, appropriately designed larger-scale studies in both CKD and ESRD cohorts are necessary to substantiate these preliminary findings and to demonstrate the potential clinical utility of targeting sympathetic overactivity to decrease the high morbidity and mortality related to HF and cardiac arrhythmias in this high-risk population.

## Author Contributions

MK, JH, and MS wrote the first draft of the manuscript. JN, LG, RC, and VM wrote sections of the manuscript. All authors contributed to the manuscript revision, read, and approved the submitted version.

## Conflict of Interest

MS has been an investigator in studies sponsored by Medtronic and has received speaker fees. The remaining authors declare that the research was conducted in the absence of any commercial or financial relationships that could be construed as a potential conflict of interest.
